# Effect of sealers on fracture resistance of endodontically treated teeth with and without smear layer removal: An *in vitro* study

**DOI:** 10.4103/0972-0707.57635

**Published:** 2009

**Authors:** Swaty Jhamb, Vineeta Nikhil, Vijay Singh

**Affiliations:** Departments of Conservative and Endodontics, Dr. H S Judge Institute of Dental Sciences and Hospital, Sector - 25, Chandigarh, India; 1 Departments of Conservative and Endodontics, D.A.V (C) Dental College, Yamunanagar, India

**Keywords:** Acroseal, EDTA, EGTA, Instron, Katac-Endo, smear layar

## Abstract

**Aim:**

The present study involved the *in vitro* comparison of root reinforcing abilities of two sealers, i.e., Ketac-Endo and Acroseal, in endodontically treated teeth in the presence and absence of smear layer.

**Materials and Methods:**

Fifty teeth were taken and sectioned at the cementoenamel junction. The teeth with faults were discarded and a total of 36 teeth were used for study. The samples were biomechanically prepared using step-back technique. In 10 teeth, the smear layer was preserved using sodium hypochlorite. Smear layers were removed from 10 teeth using 17% EDTA, and in another 10 samples, the smear layers were eliminated using 17% EGTA. The remaining samples served as controls. Samples were obturated with sealers using the lateral condensation technique. Ketac-Endo (3M) is a glass ionomer based root canal sealer, and Acroseal (Septodont) sealers were used. The teeth were then tested by using an Instron testing machine.

**Results:**

Ketac-Endo shows higher fracture resistance values in comparison to Acroseal. Other factors as the amount of tooth structure remaining, the agents used for the removal of smear layer and instrumentation techniques may alter the tooth resistance to fracture.

**Conclusion:**

Ketac-Endo shows higher fracture resistance values in comparison to Acroseal.

## INTRODUCTION

The major aim of endodontic therapy is to attain a clean root canal, which allows the three-dimensional obturation of the root canal. The strength of an endodontically treated tooth is directly related to the amount of remaining sound tooth structure. Caries removal, access preparation, canal instrumentation and preparation for final restoration all lead to loss of tooth structure, structurally weakening the tooth. The greatest incidence of vertical root fracture occurs in teeth after endodontic therapy. The reason most often cited has been the dehydration of dentin after endodontic therapy, excessive pressure during obturation, and the removal of tooth structure during endodontic therapy. Vertical root fracture at the time of canal obturation or during subsequent use of the tooth is one of the most serious complications of root canal therapy, and most of these cases require extraction of the affected tooth.[1] Clinicians have long sought to reinforce remaining tooth structure. The instrumented root canal seems to be an ideal environment for obtaining the maximal bond of the sealer. The bonding of endodontic sealers to interradicular dentin after root obturation might possibly enhance the resistance to fracture of endodontically treated teeth. The use of a root canal sealer with properties similar to those of other sealers and with the additional quality of strengthening the root against fracture would then be of value.[2]

## MATERIALS AND METHODS

For the *in vitro* study, 50 extracted maxillary central incisors from patients in the age group 40-55 years were obtained. After extraction, soft tissue and calculus were mechanically removed and teeth were stored in 5% sodium hypochlorite solution for 24 h to remove any remaining soft tissue. Certain teeth that had fracture lines, calcifications, surface irregularities were discarded, and a total of 36 teeth samples were obtained for the study. Although the sample size was small, it was sufficient to achieve a statistical difference. The teeth were sectioned at the cementoenamel junction using a diamond disc and water spray The sectioned teeth were taken, and a working length for each root was then established 1 mm short of the apical foramen using a No. 20 K-file. Further, the roots were divided randomly into three groups.

### Experimental groups

#### Group I

Ten roots were taken and a standardized technique described by Grossman was used to instrument each root canal three sizes larger than the first file that bound at the established working length. In these teeth, smear layer was left intact and irrigation was done using 15 ml of 5% sodium hypochlorite solution.

#### Group II

Ten teeth were prepared as described in Group I, and the smear layer was removed by using 10 ml of 17% EDTA solution, followed by a final irrigation with 10 ml of 5% sodium hypochlorite.

#### Group III

Ten teeth were prepared as described in Groups I and II, and the smear layer was removed by using 10 ml of 17% EGTA followed by a final irrigation with 10 ml of 5% sodium hypochlorite.

These groups were further subdivided into two subgroups (a and b) by obturation utilizing lateral condensation with Gutta percha and either Acroseal (subgroup a) or Ketac-Endo (subgroup b) The subgroups contained five teeth each.

#### Group IV (positive control)

Three roots were taken and instrumented using standardized technique but were not obturated.

#### Group V (negative control)

Three roots were taken and were neither instrumented nor obturated.

The roots were coronally sealed with Cavit. The specimens were placed in cotton moistened with sterile water to attain 100% humidity and then were placed in an incubator at 37°C for 8 days to allow the sealers to set completely.

The samples were mounted for testing in rings of a polychlorovinyl pipe with a diameter and height of 0.75 in. and 1 in., respectively. Thirty-gauge round orthodontic wire was bent into a square “J” shape. Sticky wax was used to attach the short handle of the “J” to the canal orifice of each sample and the long handle to the outer surface of the CPVC rings. This allowed suspension of the tooth in the center of the ring to be parallel to the long axis of the root. Die stone was poured into the ring to stabilize the sample so that coronal-most portion of each root projected 4 mm above the stone. The samples were then stored in 100% humidity until testing. Strength testing was accomplished by using a universal testing machine. A circular stainless steel rod with a diameter of 3 mm and beveled 45 degrees at the tip was fixed to the upper stage of Instron. The mounted teeth were placed on the lower platform on top of a 0.5-in. foam piece, which allowed for slight movement and adjustment of the samples as the force was applied so that the force would be directed vertically down the long axis of the root. The upper stage was positioned so that the rod was centered over the access opening of each tested root until the bevel of the rod just contacted the circumference of the orifice. Vertical compressive force was applied at a cross-head speed of 0.5 mm/min, and the force (in kilograms) needed to fracture each tooth was recorded.

## RESULTS

The mean fracture resistance values of Group I a were 12.88 kg, Group I b were 15.70 kg, Group II a were 17.25 kg, Group II b were 20.78 kg, Group III a were 17.94 kg, Group III b were 22.82 kg, positive control were 7.21 kg and negative control were 34.83 kg [Table 1].

### Statistical analysis

Statistical analysis was done using one way analysis of variance and unpaired student's *t*-test [Table 2]. To test the equality of several means (more than two means), analysis of variance (ANOVA) [Table 3] was used. The ‘*P*’ value was taken as significant at *P* < 0.05.

## DISCUSSION

The present study compared the fracture resistance of endodontically treated teeth. The teeth in the study were obturated using lateral condensation technique because it is a widely recommended, commonly followed technique and facilitates comparison.[3] The force in this study was directed at an angle of 0°, resulting in primarily a splitting stress applied above the access opening. The teeth had only 4 mm of root dentin exposed above the embedding material. This resulted in smaller stresses due to decreased bending movements and maximum stress located more cervically. This design is more relevant clinically as it efficiently simulates the support given to healthy teeth by alveolar bone and results in less catastrophic stress build ups caused by unrealistic bending movements.[4]

**Table 1:** Mean values of fracture resistance

Groups	Mean resistance values (kg)
I^a^	12.88
I^b^	15.70
II^a^	17.25
II^b^	20.78
III^a^	17.94
III^b^	22.82
IV	7.21
V	34.83

a = rank 1

b = rank 2

**Table 2:** Student's t-test value table

Groups	*t* values	*P* values
I^a^–II^a^	3.023	0.01648
I^a^–III^a^	2.048	0.00474
I^a^–III^b^	2.665	0.02771
I^a^–IV	2.970	0.02496
I^a^–V	3.016	0.02352
I^b^–IV	2.329	0.05872
I^b^–V	2.417	0.05208
II^a^–IV	6.248	0.0078
II^a^–V	2.439	0.05054
II^b^–IV	2.353	0.05682
II^b^–V	1.545	0.01733
III^a^–IV	3.439	0.01382
III^a^–V	2.196	0.00704
III^b^–IV	3.239	0.0177
IV–V	2.802	0.04871

a = rank 1

b = rank 2

**Table 3:** Analysis of variance table for fracture resistance testing

Source of variation	Degree of freedom	Variance ratio	*P* value
Between groups	4	6.87821	0.000438
Within group	31	-	-
2TCL:Total	35	-	-

The groups that used Ketac-Endo as a sealer had higher values of fracture resistance. Higher values of Ketac-Endo may be explained on the basis of the fact that Ketac-Endo forms a chemical bond with the root dentin. This chemical bond prevents percolation and bacterial penetration.[2] Certain authors disagree with the above findings and suggest that root reinforcement through bonded sealers was largely due to a small ratio of reinforcing material to root surface and not due to its bonding capability.[1] Moreover, may be the thickness of the sealer decreased during lateral condensation, and that is why Ketac-Endo sealed more tightly.[5] However, a variation was recorded in the readings within the Ketac-Endo group. This may be because Ketac-Endo is a technique-sensitive material. Excessive moisture or drying results in a loss of bond between the material and tooth structure.[1] Another possible reason for variation in fracture load and poor correlation of fracture loads to size and taper of root canal could be difference in calcification of individual teeth as well as surfaces irregularities, developmental grooves and nonuniformity of canal walls. These irregularities could lead to high stress concentration in the roots.[6]

Samples in which smear layer was preserved using sodium hypochlorite showed lower values of fracture resistance. This may be because 5% sodium hypochlorite depletes the organic phase and causes a mechanical change by release of hypochlorous acid, which reacts with insoluble proteins to form soluble polypeptides, amino acids and other byproducts. The results are in agreement with other previous studies. [7–9] It has been shown that the presence of smear layer reduces root dentin permeability by 25%. Sodium hypochlorite also causes a decrease in hardness. The decrease may be because of the decrease in the stiffness of intertubular dentin matrix caused by heterogeneous distribution of mineral phase within the collagen matrix.[10]

Roots in which the smear layer was removed using 17% EDTA required more loads to fracture them. This may be due to the demineralizing ability of 17% EDTA and also its ability to remove the inorganic components of smear layer. 17% EDTA has been shown to solubilize the highest percentage, which is greater than 70%, of the inorganic portion of dentin. [11] This may have allowed a greater adhesion of Acroseal as well as Ketac-Endo to root dentin, thereby strengthening of the endodontically treated teeth. 17% EDTA removes the smear layer up to a depth of 2.5-4 μm. Disodium salt of 17% EDTA is generally accepted as the most effective chelating agent with prominent lubricant properties and is widely used in endodontic therapy. [12] Moreover, EDTA has a low surface tension, which allows it to easily flow into the dentinal tubules.[13] After the removal of smear layer, there was an alteration in the surface energy allowing the sealer to flow and adapt more easily, enhancing its adhesion. However, certain authors have stated that there was no difference among the root fracture resistance of sealers, regardless of the absence or presence of smear layer.[14]

Samples in which the smear layer was removed using 17% EGTA showed higher values of fracture resistance than that of groups using 17% EDTA and also those in which the smear layer was preserved. The higher values may be explained on the basis of the following: 17% EGTA opens a greater number of dentinal tubules than 17% EDTA[15] without inducing an erosive action. Moreover, 17% EGTA causes chelation of only calcium ions, whereas17% EDTA chelates calcium ions along with magnesium ions causing a slight increase in embrittlement.[16]

Teeth that were instrumented but not obturated showed vertical fracture at very less loads in comparison to other experimental groups and the results were statistically significant (*P* < 0.05). This may be due to the fact that root canal instrumentation weakened the roots as the amount of remaining dentin thickness was greatly reduced, and there was no obturating material to provide strength.[17] Mechanical instrumentation of root canals can produce craze lines on root canal wall, which may serve as localized sites of increased stress. [18] The larger the preparation size, the greater is the strength loss. Moreover, the amount of remaining dentin thickness significantly affected the resistance to fracture of the prepared roots.[2,17,19]

Teeth that were neither biomechanically prepared nor obturated had the highest scores of fracture resistance and the results were statistically significant (*P* < 0.05). The reason may be that root canal preparation weakened the root canals, and significant strength could not be attained after obturation.[1]

A large sample size along with clinical trials is necessary to validate the results of the present *in vitro* study.

## CONCLUSIONS

The present study clearly states that:

1. Roots were significantly weakened after instrumentation.
2. Ketac-Endo samples exhibited better results but there was no significant difference among root fracture resistance of Ketac-Endo and Acroseal sealer, regardless of the absence or presence of smear layer.
3. EGTA is a more potent smear layer removing agent in comparison to normally used EDTA.
4. The roots that were obturated with two sealers and lateral condensation technique were significantly stronger than the roots whose canals were instrumented but not obturated.
